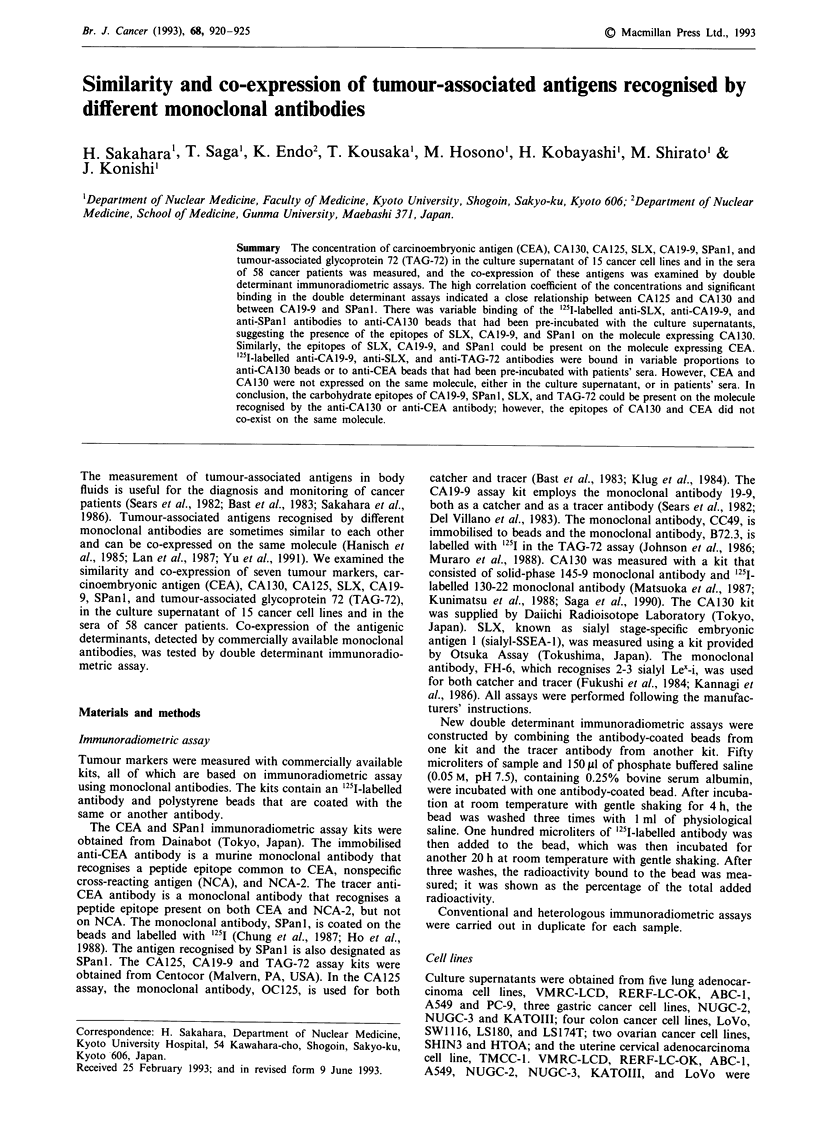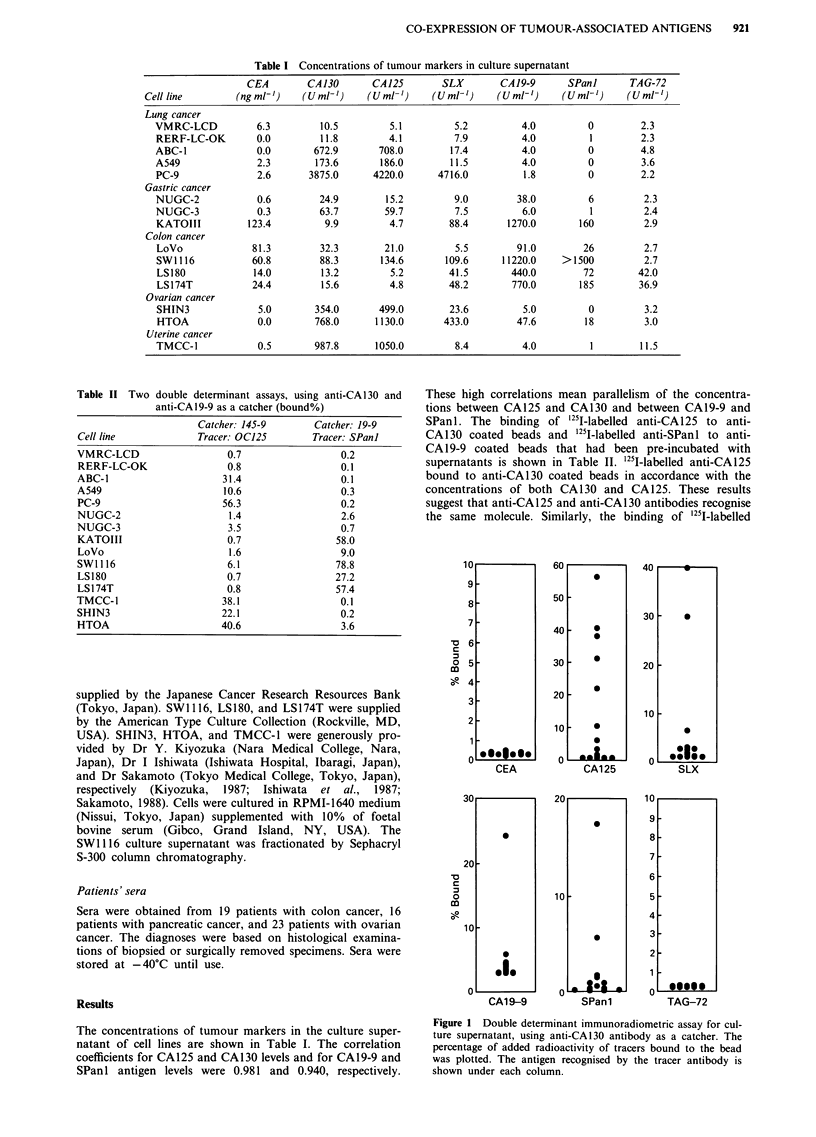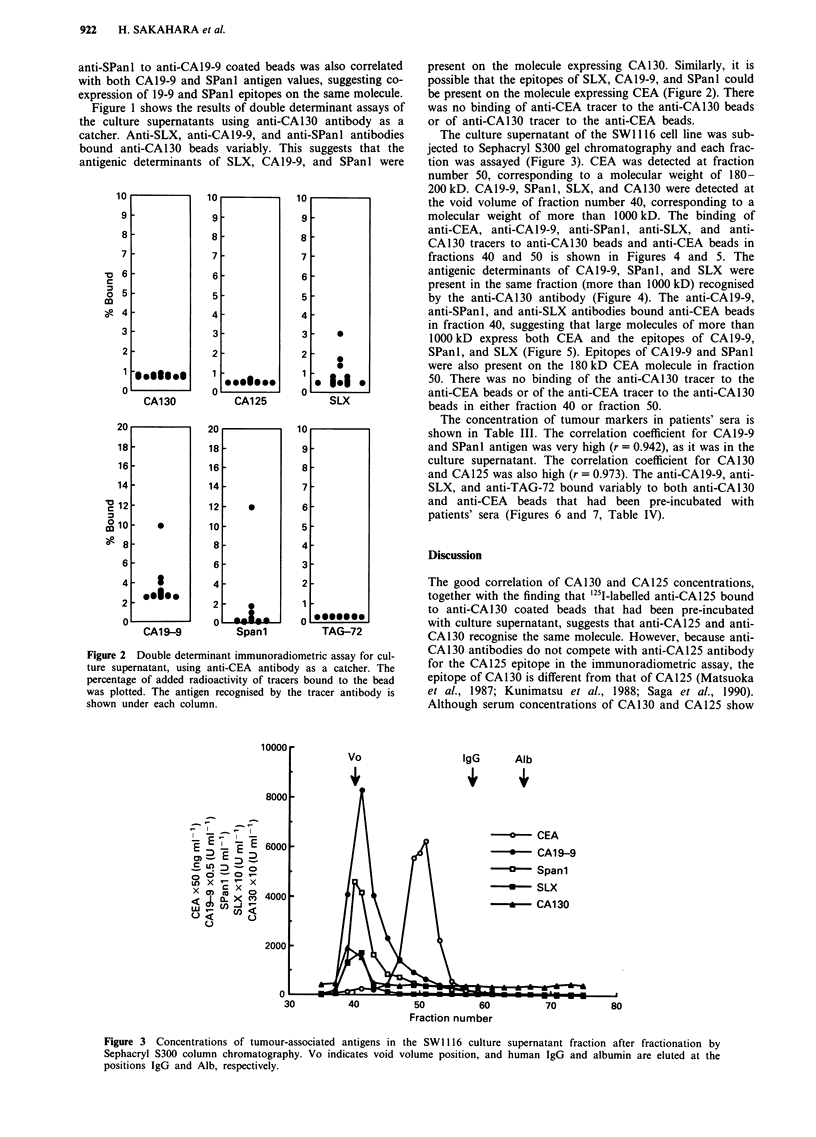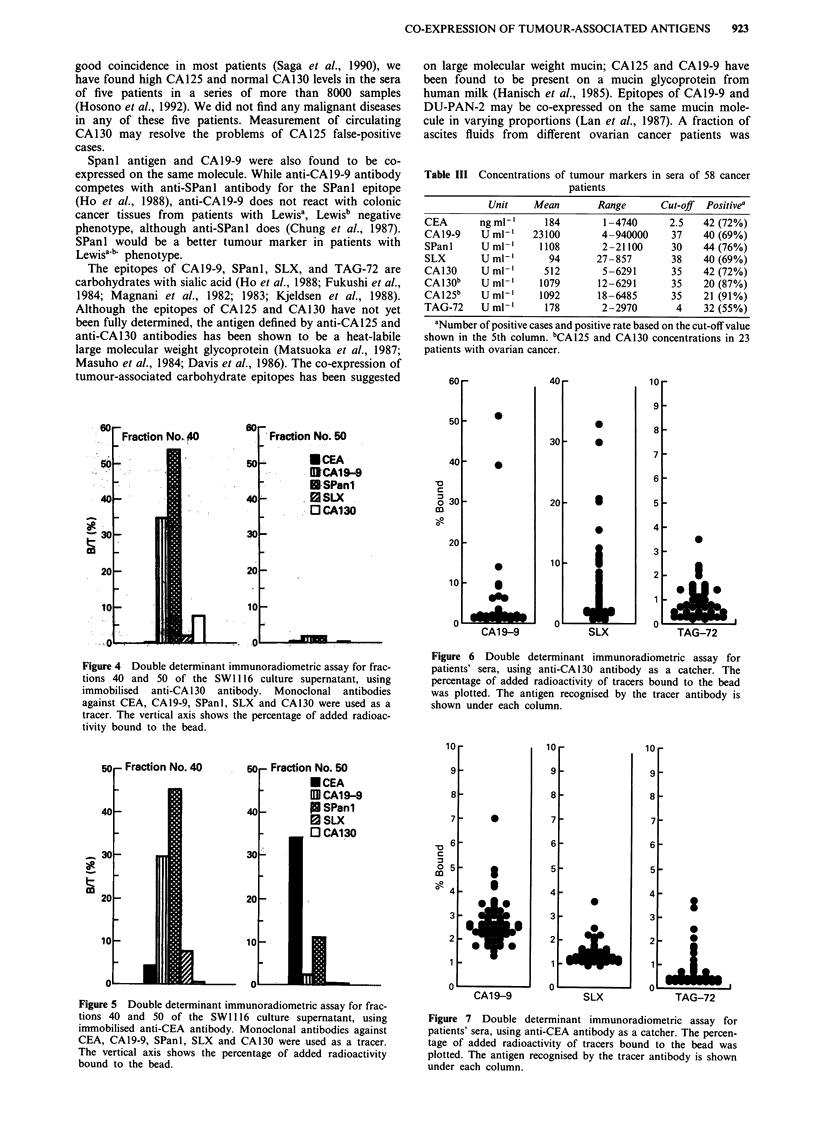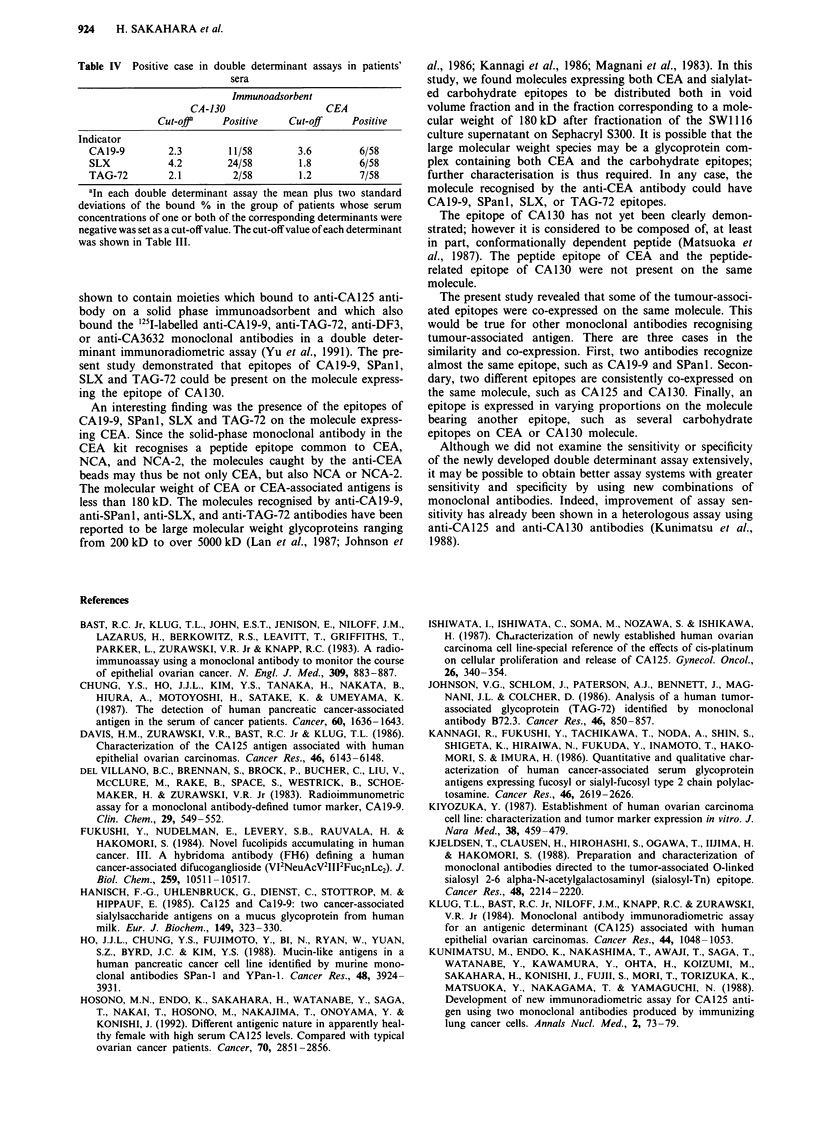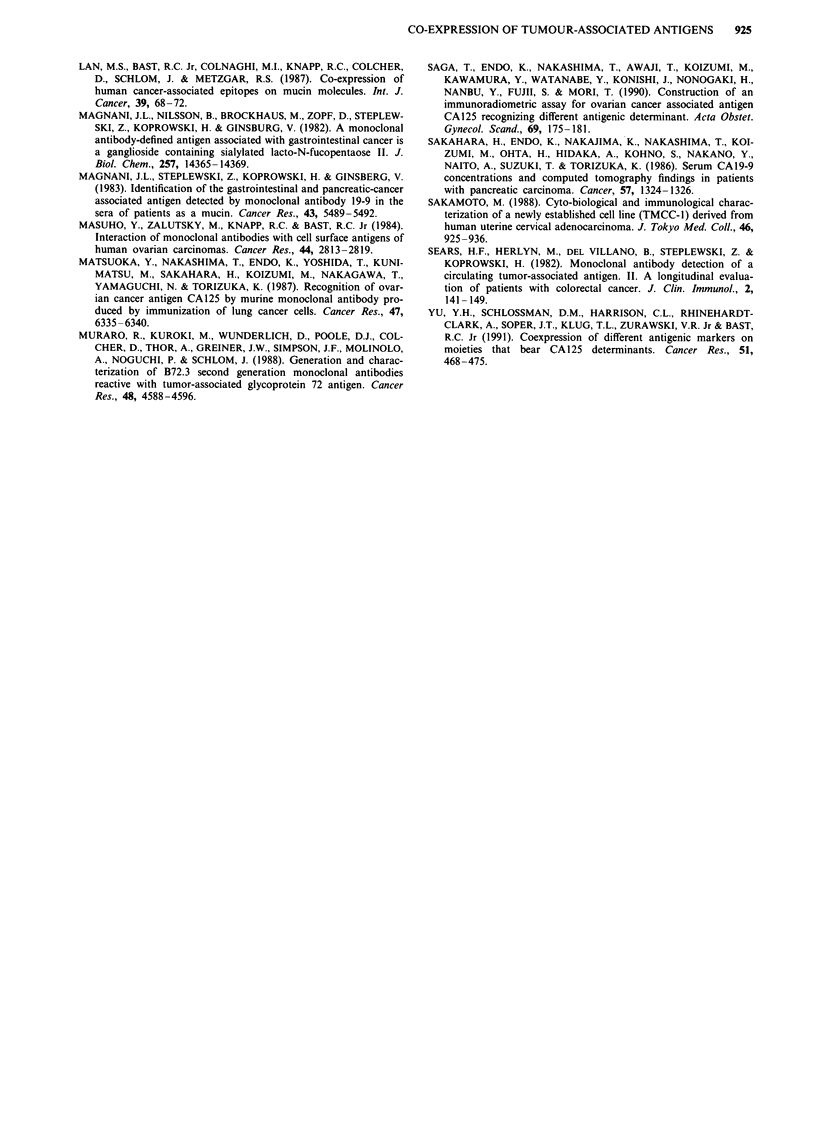# Similarity and co-expression of tumour-associated antigens recognised by different monoclonal antibodies.

**DOI:** 10.1038/bjc.1993.455

**Published:** 1993-11

**Authors:** H. Sakahara, T. Saga, K. Endo, T. Kousaka, M. Hosono, H. Kobayashi, M. Shirato, J. Konishi

**Affiliations:** Department of Nuclear Medicine, Faculty of Medicine, Kyoto University, Japan.

## Abstract

The concentration of carcinoembryonic antigen (CEA), CA130, CA125, SLX, CA19-9, SPan1, and tumour-associated glycoprotein 72 (TAG-72) in the culture supernatant of 15 cancer cell lines and in the sera of 58 cancer patients was measured, and the co-expression of these antigens was examined by double determinant immunoradiometric assays. The high correlation coefficient of the concentrations and significant binding in the double determinant assays indicated a close relationship between CA125 and CA130 and between CA19-9 and SPan1. There was variable binding of the 125I-labelled anti-SLX, anti-CA19-9, and anti-SPan1 antibodies to anti-CA130 beads that had been pre-incubated with the culture supernatants, suggesting the presence of the epitopes of SLX, CA19-9, and SPan1 on the molecule expressing CA130. Similarly, the epitopes of SLX, CA19-9, and SPan1 could be present on the molecule expressing CEA. 125I-labelled anti-CA19-9, anti-SLX, and anti-TAG-72 antibodies were bound in variable proportions to anti-CA130 beads or to anti-CEA beads that had been pre-incubated with patients' sera. However, CEA and CA130 were not expressed on the same molecule, either in the culture supernatant, or in patients' sera. In conclusion, the carbohydrate epitopes of CA19-9, SPan1, SLX, and TAG-72 could be present on the molecule recognised by the anti-CA130 or anti-CEA antibody; however, the epitopes of CA130 and CEA did not co-exist on the same molecule.


Br. J. Cancer (1993), 68, 920-925 © Macmillan Press Ltd., 1993

### Similarity and co-expression of tumour-associated antigens recognised by different monoclonal antibodies

H. Sakahara[1], T. Saga[1], K. Endo[2], T. Kousaka[1], M. Hosono[1], H. Kobayashi[1], M. Shirato[1] & J. Konishi[1]

[1]*Department of Nuclear Medicine, Faculty of Medicine, Kyoto University, Shogoin, Sakyo-ku, Kyoto 606;* [2]*Department of Nuclear Medicine, School of Medicine, Gunma University, Maebashi 371, Japan.*

**Summary** The concentration of carcinoembryonic antigen (CEA), CA130, CA125, SLX, CA19-9, SPan1, and tumour-associated glycoprotein 72 (TAG-72) in the culture supernatant of 15 cancer cell lines and in the sera of 58 cancer patients was measured, and the co-expression of these antigens was examined by double determinant immunoradiometric assays. The high correlation coefficient of the concentrations and significant binding in the double determinant assays indicated a close relationship between CA125 and CA130 and between CA19-9 and SPan1. There was variable binding of the $^{125}$I-labelled anti-SLX, anti-CA19-9, and anti-SPan1 antibodies to anti-CA130 beads that had been pre-incubated with the culture supernatants, suggesting the presence of the epitopes of SLX, CA19-9, and SPan1 on the molecule expressing CA130. Similarly, the epitopes of SLX, CA19-9, and SPan1 could be present on the molecule expressing CEA. $^{125}$I-labelled anti-CA19-9, anti-SLX, and anti-TAG-72 antibodies were bound in variable proportions to anti-CA130 beads or to anti-CEA beads that had been pre-incubated with patients' sera. However, CEA and CA130 were not expressed on the same molecule, either in the culture supernatant, or in patients' sera. In conclusion, the carbohydrate epitopes of CA19-9, SPan1, SLX, and TAG-72 could be present on the molecule recognised by the anti-CA130 or anti-CEA antibody; however, the epitopes of CA130 and CEA did not co-exist on the same molecule.

The measurement of tumour-associated antigens in body fluids is useful for the diagnosis and monitoring of cancer patients (Sears *et al.*, 1982; Bast *et al.*, 1983; Sakahara *et al.*, 1986). Tumour-associated antigens recognised by different monoclonal antibodies are sometimes similar to each other and can be co-expressed on the same molecule (Hanisch *et al.*, 1985; Lan *et al.*, 1987; Yu *et al.*, 1991). We examined the similarity and co-expression of seven tumour markers, carcinoembryonic antigen (CEA), CA130, CA125, SLX, CA19-9, SPan1, and tumour-associated glycoprotein 72 (TAG-72), in the culture supernatant of 15 cancer cell lines and in the sera of 58 cancer patients. Co-expression of the antigenic determinants, detected by commercially available monoclonal antibodies, was tested by double determinant immunoradiometric assay.

### Materials and methods

### *Immunoradiometric assay*

Tumour markers were measured with commercially available kits, all of which are based on immunoradiometric assay using monoclonal antibodies. The kits contain an $^{125}$I-labelled antibody and polystyrene beads that are coated with the same or another antibody.

The CEA and SPan1 immunoradiometric assay kits were obtained from Dainabot (Tokyo, Japan). The immobilised anti-CEA antibody is a murine monoclonal antibody that recognises a peptide epitope common to CEA, nonspecific cross-reacting antigen (NCA), and NCA-2. The tracer anti-CEA antibody is a monoclonal antibody that recognises a peptide epitope present on both CEA and NCA-2, but not on NCA. The monoclonal antibody, SPan1, is coated on the beads and labelled with $^{125}$I (Chung *et al.*, 1987; Ho *et al.*, 1988). The antigen recognised by SPan1 is also designated as SPan1. The CA125, CA19-9 and TAG-72 assay kits were obtained from Centocor (Malvern, PA, USA). In the CA125 assay, the monoclonal antibody, OC125, is used for both catcher and tracer (Bast *et al.*, 1983; Klug *et al.*, 1984). The CA19-9 assay kit employs the monoclonal antibody 19-9, both as a catcher and as a tracer antibody (Sears *et al.*, 1982; Del Villano *et al.*, 1983). The monoclonal antibody, CC49, is immobilised to beads and the monoclonal antibody, B72.3, is labelled with $^{125}$I in the TAG-72 assay (Johnson *et al.*, 1986; Muraro *et al.*, 1988). CA130 was measured with a kit that consisted of solid-phase 145-9 monoclonal antibody and $^{125}$I-labelled 130-22 monoclonal antibody (Matsuoka *et al.*, 1987; Kunimatsu *et al.*, 1988; Saga *et al.*, 1990). The CA130 kit was supplied by Daiichi Radioisotope Laboratory (Tokyo, Japan). SLX, known as sialyl stage-specific embryonic antigen 1 (sialyl-SSEA-1), was measured using a kit provided by Otsuka Assay (Tokushima, Japan). The monoclonal antibody, FH-6, which recognises 2-3 sialyl $Le^x$-i, was used for both catcher and tracer (Fukushi *et al.*, 1984; Kannagi *et al.*, 1986). All assays were performed following the manufacturers' instructions.

New double determinant immunoradiometric assays were constructed by combining the antibody-coated beads from one kit and the tracer antibody from another kit. Fifty microliters of sample and 150 µl of phosphate buffered saline (0.05 M, pH 7.5), containing 0.25% bovine serum albumin, were incubated with one antibody-coated bead. After incubation at room temperature with gentle shaking for 4 h, the bead was washed three times with 1 ml of physiological saline. One hundred microliters of $^{125}$I-labelled antibody was then added to the bead, which was then incubated for another 20 h at room temperature with gentle shaking. After three washes, the radioactivity bound to the bead was measured; it was shown as the percentage of the total added radioactivity.

Conventional and heterologous immunoradiometric assays were carried out in duplicate for each sample.

### *Cell lines*

Culture supernatants were obtained from five lung adenocarcinoma cell lines, VMRC-LCD, RERF-LC-OK, ABC-1, A549 and PC-9, three gastric cancer cell lines, NUGC-2, NUGC-3 and KATOIII; four colon cancer cell lines, LoVo, SW1116, LS180, and LS174T; two ovarian cancer cell lines, SHIN3 and HTOA; and the uterine cervical adenocarcinoma cell line, TMCC-1. VMRC-LCD, RERF-LC-OK, ABC-1, A549, NUGC-2, NUGC-3, KATOIII, and LoVo were

Correspondence: H. Sakahara, Department of Nuclear Medicine, Kyoto University Hospital, 54 Kawahara-cho, Shogoin, Sakyo-ku, Kyoto 606, Japan.
Received 25 February 1993; and in revised form 9 June 1993.

**Table I** Concentrations of tumour markers in culture supernatant

| *Cell line* | *CEA* (*ng ml*$^{-1}$) | *CA130* (*U ml*$^{-1}$) | *CA125* (*U ml*$^{-1}$) | *SLX* (*U ml*$^{-1}$) | *CA19-9* (*U ml*$^{-1}$) | *SPan1* (*U ml*$^{-1}$) | *TAG-72* (*U ml*$^{-1}$) |
|---|---|---|---|---|---|---|---|
| *Lung cancer* | | | | | | | |
| VMRC-LCD | 6.3 | 10.5 | 5.1 | 5.2 | 4.0 | 0 | 2.3 |
| RERF-LC-OK | 0.0 | 11.8 | 4.1 | 7.9 | 4.0 | 1 | 2.3 |
| ABC-1 | 0.0 | 672.9 | 708.0 | 17.4 | 4.0 | 0 | 4.8 |
| A549 | 2.3 | 173.6 | 186.0 | 11.5 | 4.0 | 0 | 3.6 |
| PC-9 | 2.6 | 3875.0 | 4220.0 | 4716.0 | 1.8 | 0 | 2.2 |
| *Gastric cancer* | | | | | | | |
| NUGC-2 | 0.6 | 24.9 | 15.2 | 9.0 | 38.0 | 6 | 2.3 |
| NUGC-3 | 0.3 | 63.7 | 59.7 | 7.5 | 6.0 | 1 | 2.4 |
| KATOIII | 123.4 | 9.9 | 4.7 | 88.4 | 1270.0 | 160 | 2.9 |
| *Colon cancer* | | | | | | | |
| LoVo | 81.3 | 32.3 | 21.0 | 5.5 | 91.0 | 26 | 2.7 |
| SW1116 | 60.8 | 88.3 | 134.6 | 109.6 | 11220.0 | >1500 | 2.7 |
| LS180 | 14.0 | 13.2 | 5.2 | 41.5 | 440.0 | 72 | 42.0 |
| LS174T | 24.4 | 15.6 | 4.8 | 48.2 | 770.0 | 185 | 36.9 |
| *Ovarian cancer* | | | | | | | |
| SHIN3 | 5.0 | 354.0 | 499.0 | 23.6 | 5.0 | 0 | 3.2 |
| HTOA | 0.0 | 768.0 | 1130.0 | 433.0 | 47.6 | 18 | 3.0 |
| *Uterine cancer* | | | | | | | |
| TMCC-1 | 0.5 | 987.8 | 1050.0 | 8.4 | 4.0 | 1 | 11.5 |

**Table II** Two double determinant assays, using anti-CA130 and anti-CA19-9 as a catcher (bound%)

| *Cell line* | *Catcher: 145-9* *Tracer: OC125* | *Catcher: 19-9* *Tracer: SPan1* |
|---|---|---|
| VMRC-LCD | 0.7 | 0.2 |
| RERF-LC-OK | 0.8 | 0.1 |
| ABC-1 | 31.4 | 0.1 |
| A549 | 10.6 | 0.3 |
| PC-9 | 56.3 | 0.2 |
| NUGC-2 | 1.4 | 2.6 |
| NUGC-3 | 3.5 | 0.7 |
| KATOIII | 0.7 | 58.0 |
| LoVo | 1.6 | 9.0 |
| SW1116 | 6.1 | 78.8 |
| LS180 | 0.7 | 27.2 |
| LS174T | 0.8 | 57.4 |
| TMCC-1 | 38.1 | 0.1 |
| SHIN3 | 22.1 | 0.2 |
| HTOA | 40.6 | 3.6 |

supplied by the Japanese Cancer Research Resources Bank (Tokyo, Japan). SW1116, LS180, and LS174T were supplied by the American Type Culture Collection (Rockville, MD, USA). SHIN3, HTOA, and TMCC-1 were generously provided by Dr Y. Kiyozuka (Nara Medical College, Nara, Japan), Dr I Ishiwata (Ishiwata Hospital, Ibaragi, Japan), and Dr Sakamoto (Tokyo Medical College, Tokyo, Japan), respectively (Kiyozuka, 1987; Ishiwata *et al.*, 1987; Sakamoto, 1988). Cells were cultured in RPMI-1640 medium (Nissui, Tokyo, Japan) supplemented with 10% of foetal bovine serum (Gibco, Grand Island, NY, USA). The SW1116 culture supernatant was fractionated by Sephacryl S-300 column chromatography.

### *Patients' sera*

Sera were obtained from 19 patients with colon cancer, 16 patients with pancreatic cancer, and 23 patients with ovarian cancer. The diagnoses were based on histological examinations of biopsied or surgically removed specimens. Sera were stored at −40°C until use.

### Results

The concentrations of tumour markers in the culture supernatant of cell lines are shown in Table I. The correlation coefficients for CA125 and CA130 levels and for CA19-9 and SPan1 antigen levels were 0.981 and 0.940, respectively. These high correlations mean parallelism of the concentrations between CA125 and CA130 and between CA19-9 and SPan1. The binding of $^{125}$I-labelled anti-CA125 to anti-CA130 coated beads and $^{125}$I-labelled anti-SPan1 to anti-CA19-9 coated beads that had been pre-incubated with supernatants is shown in Table II. $^{125}$I-labelled anti-CA125 bound to anti-CA130 coated beads in accordance with the concentrations of both CA130 and CA125. These results suggest that anti-CA125 and anti-CA130 antibodies recognise the same molecule. Similarly, the binding of $^{125}$I-labelled

**Figure 1** Double determinant immunoradiometric assay for culture supernatant, using anti-CA130 antibody as a catcher. The percentage of added radioactivity of tracers bound to the bead was plotted. The antigen recognised by the tracer antibody is shown under each column.

anti-SPan1 to anti-CA19-9 coated beads was also correlated with both CA19-9 and SPan1 antigen values, suggesting co-expression of 19-9 and SPan1 epitopes on the same molecule.

Figure 1 shows the results of double determinant assays of the culture supernatants using anti-CA130 antibody as a catcher. Anti-SLX, anti-CA19-9, and anti-SPan1 antibodies bound anti-CA130 beads variably. This suggests that the antigenic determinants of SLX, CA19-9, and SPan1 were present on the molecule expressing CA130. Similarly, it is possible that the epitopes of SLX, CA19-9, and SPan1 could be present on the molecule expressing CEA (Figure 2). There was no binding of anti-CEA tracer to the anti-CA130 beads or of anti-CA130 tracer to the anti-CEA beads.

**Figure 2** Double determinant immunoradiometric assay for culture supernatant, using anti-CEA antibody as a catcher. The percentage of added radioactivity of tracers bound to the bead was plotted. The antigen recognised by the tracer antibody is shown under each column.

The culture supernatant of the SW1116 cell line was subjected to Sephacryl S300 gel chromatography and each fraction was assayed (Figure 3). CEA was detected at fraction number 50, corresponding to a molecular weight of 180–200 kD. CA19-9, SPan1, SLX, and CA130 were detected at the void volume of fraction number 40, corresponding to a molecular weight of more than 1000 kD. The binding of anti-CEA, anti-CA19-9, anti-SPan1, anti-SLX, and anti-CA130 tracers to anti-CA130 beads and anti-CEA beads in fractions 40 and 50 is shown in Figures 4 and 5. The antigenic determinants of CA19-9, SPan1, and SLX were present in the same fraction (more than 1000 kD) recognised by the anti-CA130 antibody (Figure 4). The anti-CA19-9, anti-SPan1, and anti-SLX antibodies bound anti-CEA beads in fraction 40, suggesting that large molecules of more than 1000 kD express both CEA and the epitopes of CA19-9, SPan1, and SLX (Figure 5). Epitopes of CA19-9 and SPan1 were also present on the 180 kD CEA molecule in fraction 50. There was no binding of the anti-CA130 tracer to the anti-CEA beads or of the anti-CEA tracer to the anti-CA130 beads in either fraction 40 or fraction 50.

The concentration of tumour markers in patients' sera is shown in Table III. The correlation coefficient for CA19-9 and SPan1 antigen was very high ($r = 0.942$), as it was in the culture supernatant. The correlation coefficient for CA130 and CA125 was also high ($r = 0.973$). The anti-CA19-9, anti-SLX, and anti-TAG-72 bound variably to both anti-CA130 and anti-CEA beads that had been pre-incubated with patients' sera (Figures 6 and 7, Table IV).

### Discussion

The good correlation of CA130 and CA125 concentrations, together with the finding that $^{125}$I-labelled anti-CA125 bound to anti-CA130 coated beads that had been pre-incubated with culture supernatant, suggests that anti-CA125 and anti-CA130 recognise the same molecule. However, because anti-CA130 antibodies do not compete with anti-CA125 antibody for the CA125 epitope in the immunoradiometric assay, the epitope of CA130 is different from that of CA125 (Matsuoka *et al.*, 1987; Kunimatsu *et al.*, 1988; Saga *et al.*, 1990). Although serum concentrations of CA130 and CA125 show

**Figure 3** Concentrations of tumour-associated antigens in the SW1116 culture supernatant fraction after fractionation by Sephacryl S300 column chromatography. Vo indicates void volume position, and human IgG and albumin are eluted at the positions IgG and Alb, respectively.

good coincidence in most patients (Saga *et al.*, 1990), we have found high CA125 and normal CA130 levels in the sera of five patients in a series of more than 8000 samples (Hosono *et al.*, 1992). We did not find any malignant diseases in any of these five patients. Measurement of circulating CA130 may resolve the problems of CA125 false-positive cases.

Spanl antigen and CA19-9 were also found to be co-expressed on the same molecule. While anti-CA19-9 antibody competes with anti-SPanl antibody for the SPanl epitope (Ho *et al.*, 1988), anti-CA19-9 does not react with colonic cancer tissues from patients with Lewis[a], Lewis[b] negative phenotype, although anti-SPanl does (Chung *et al.*, 1987). SPanl would be a better tumour marker in patients with Lewis[a-b-] phenotype.

The epitopes of CA19-9, SPanl, SLX, and TAG-72 are carbohydrates with sialic acid (Ho *et al.*, 1988; Fukushi *et al.*, 1984; Magnani *et al.*, 1982; 1983; Kjeldsen *et al.*, 1988). Although the epitopes of CA125 and CA130 have not yet been fully determined, the antigen defined by anti-CA125 and anti-CA130 antibodies has been shown to be a heat-labile large molecular weight glycoprotein (Matsuoka *et al.*, 1987; Masuho *et al.*, 1984; Davis *et al.*, 1986). The co-expression of tumour-associated carbohydrate epitopes has been suggested on large molecular weight mucin; CA125 and CA19-9 have been found to be present on a mucin glycoprotein from human milk (Hanisch *et al.*, 1985). Epitopes of CA19-9 and DU-PAN-2 may be co-expressed on the same mucin molecule in varying proportions (Lan *et al.*, 1987). A fraction of ascites fluids from different ovarian cancer patients was

**Figure 4** Double determinant immunoradiometric assay for fractions 40 and 50 of the SW1116 culture supernatant, using immobilised anti-CA130 antibody. Monoclonal antibodies against CEA, CA19-9, SPanl, SLX and CA130 were used as a tracer. The vertical axis shows the percentage of added radioactivity bound to the bead.

**Figure 5** Double determinant immunoradiometric assay for fractions 40 and 50 of the SW1116 culture supernatant, using immobilised anti-CEA antibody. Monoclonal antibodies against CEA, CA19-9, SPanl, SLX and CA130 were used as a tracer. The vertical axis shows the percentage of added radioactivity bound to the bead.

**Table III** Concentrations of tumour markers in sera of 58 cancer patients

| | *Unit* | *Mean* | *Range* | *Cut-off* | *Positive*[a] |
|---|---|---|---|---|---|
| CEA | ng ml$^{-1}$ | 184 | 1–4740 | 2.5 | 42 (72%) |
| CA19-9 | U ml$^{-1}$ | 23100 | 4–940000 | 37 | 40 (69%) |
| SPanl | U ml$^{-1}$ | 1108 | 2–21100 | 30 | 44 (76%) |
| SLX | U ml$^{-1}$ | 94 | 27–857 | 38 | 40 (69%) |
| CA130 | U ml$^{-1}$ | 512 | 5–6291 | 35 | 42 (72%) |
| CA130[b] | U ml$^{-1}$ | 1079 | 12–6291 | 35 | 20 (87%) |
| CA125[b] | U ml$^{-1}$ | 1092 | 18–6485 | 35 | 21 (91%) |
| TAG-72 | U ml$^{-1}$ | 178 | 2–2970 | 4 | 32 (55%) |

[a]Number of positive cases and positive rate based on the cut-off value shown in the 5th column. [b]CA125 and CA130 concentrations in 23 patients with ovarian cancer.

**Figure 6** Double determinant immunoradiometric assay for patients' sera, using anti-CA130 antibody as a catcher. The percentage of added radioactivity of tracers bound to the bead was plotted. The antigen recognised by the tracer antibody is shown under each column.

**Figure 7** Double determinant immunoradiometric assay for patients' sera, using anti-CEA antibody as a catcher. The percentage of added radioactivity of tracers bound to the bead was plotted. The antigen recognised by the tracer antibody is shown under each column.

**Table IV** Positive case in double determinant assays in patients' sera

| | *Immunoadsorbent* | | | |
|---|---|---|---|---|
| | *CA-130* | | *CEA* | |
| | *Cut-off*[a] | *Positive* | *Cut-off* | *Positive* |
| Indicator | | | | |
| CA19-9 | 2.3 | 11/58 | 3.6 | 6/58 |
| SLX | 4.2 | 24/58 | 1.8 | 6/58 |
| TAG-72 | 2.1 | 2/58 | 1.2 | 7/58 |

[a]In each double determinant assay the mean plus two standard deviations of the bound % in the group of patients whose serum concentrations of one or both of the corresponding determinants were negative was set as a cut-off value. The cut-off value of each determinant was shown in Table III.

shown to contain moieties which bound to anti-CA125 antibody on a solid phase immunoadsorbent and which also bound the $^{125}$I-labelled anti-CA19-9, anti-TAG-72, anti-DF3, or anti-CA3632 monoclonal antibodies in a double determinant immunoradiometric assay (Yu *et al.*, 1991). The present study demonstrated that epitopes of CA19-9, SPanl, SLX and TAG-72 could be present on the molecule expressing the epitope of CA130.

An interesting finding was the presence of the epitopes of CA19-9, SPanl, SLX and TAG-72 on the molecule expressing CEA. Since the solid-phase monoclonal antibody in the CEA kit recognises a peptide epitope common to CEA, NCA, and NCA-2, the molecules caught by the anti-CEA beads may thus be not only CEA, but also NCA or NCA-2. The molecular weight of CEA or CEA-associated antigens is less than 180 kD. The molecules recognised by anti-CA19-9, anti-SPan1, anti-SLX, and anti-TAG-72 antibodies have been reported to be large molecular weight glycoproteins ranging from 200 kD to over 5000 kD (Lan *et al.*, 1987; Johnson *et al.*, 1986; Kannagi *et al.*, 1986; Magnani *et al.*, 1983). In this study, we found molecules expressing both CEA and sialylated carbohydrate epitopes to be distributed both in void volume fraction and in the fraction corresponding to a molecular weight of 180 kD after fractionation of the SW1116 culture supernatant on Sephacryl S300. It is possible that the large molecular weight species may be a glycoprotein complex containing both CEA and the carbohydrate epitopes; further characterisation is thus required. In any case, the molecule recognised by the anti-CEA antibody could have CA19-9, SPanl, SLX, or TAG-72 epitopes.

The epitope of CA130 has not yet been clearly demonstrated; however it is considered to be composed of, at least in part, conformationally dependent peptide (Matsuoka *et al.*, 1987). The peptide epitope of CEA and the peptide-related epitope of CA130 were not present on the same molecule.

The present study revealed that some of the tumour-associated epitopes were co-expressed on the same molecule. This would be true for other monoclonal antibodies recognising tumour-associated antigen. There are three cases in the similarity and co-expression. First, two antibodies recognize almost the same epitope, such as CA19-9 and SPanl. Secondary, two different epitopes are consistently co-expressed on the same molecule, such as CA125 and CA130. Finally, an epitope is expressed in varying proportions on the molecule bearing another epitope, such as several carbohydrate epitopes on CEA or CA130 molecule.

Although we did not examine the sensitivity or specificity of the newly developed double determinant assay extensively, it may be possible to obtain better assay systems with greater sensitivity and specificity by using new combinations of monoclonal antibodies. Indeed, improvement of assay sensitivity has already been shown in a heterologous assay using anti-CA125 and anti-CA130 antibodies (Kunimatsu *et al.*, 1988).

### References

BAST, R.C. Jr, KLUG, T.L., JOHN, E.S.T., JENISON, E., NILOFF, J.M., LAZARUS, H., BERKOWITZ, R.S., LEAVITT, T., GRIFFITHS, T., PARKER, L., ZURAWSKI, V.R. Jr & KNAPP, R.C. (1983). A radioimmunoassay using a monoclonal antibody to monitor the course of epithelial ovarian cancer. *N. Engl. J. Med.*, **309**, 883–887.

CHUNG, Y.S., HO, J.J.L., KIM, Y.S., TANAKA, H., NAKATA, B., HIURA, A., MOTOYOSHI, H., SATAKE, K. & UMEYAMA, K. (1987). The detection of human pancreatic cancer-associated antigen in the serum of cancer patients. *Cancer*, **60**, 1636–1643.

DAVIS, H.M., ZURAWSKI, V.R., BAST, R.C. Jr & KLUG, T.L. (1986). Characterization of the CA125 antigen associated with human epithelial ovarian carcinomas. *Cancer Res.*, **46**, 6143–6148.

DEL VILLANO, B.C., BRENNAN, S., BROCK, P., BUCHER, C., LIU, V., MCCLURE, M., RAKE, B., SPACE, S., WESTRICK, B., SCHOEMAKER, H. & ZURAWSKI, V.R. Jr (1983). Radioimmunometric assay for a monoclonal antibody-defined tumor marker, CA19-9. *Clin. Chem.*, **29**, 549–552.

FUKUSHI, Y., NUDELMAN, E., LEVERY, S.B., RAUVALA, H. & HAKOMORI, S. (1984). Novel fucolipids accumulating in human cancer. III. A hybridoma antibody (FH6) defining a human cancer-associated difucoganglioside ($VI^2NeuAcV^2III^2Fuc_2nLc_2$). *J. Biol. Chem.*, **259**, 10511–10517.

HANISCH, F.-G., UHLENBRUCK, G., DIENST, C., STOTTROP, M. & HIPPAUF, E. (1985). Ca125 and Ca19-9: two cancer-associated sialylsaccharide antigens on a mucus glycoprotein from human milk. *Eur. J. Biochem.*, **149**, 323–330.

HO, J.J.L., CHUNG, Y.S., FUJIMOTO, Y., BI, N., RYAN, W., YUAN, S.Z., BYRD, J.C. & KIM, Y.S. (1988). Mucin-like antigens in a human pancreatic cancer cell line identified by murine monoclonal antibodies SPan-1 and YPan-1. *Cancer Res.*, **48**, 3924–3931.

HOSONO, M.N., ENDO, K., SAKAHARA, H., WATANABE, Y., SAGA, T., NAKAI, T., HOSONO, M., NAKAJIMA, T., ONOYAMA, Y. & KONISHI, J. (1992). Different antigenic nature in apparently healthy female with high serum CA125 levels. Compared with typical ovarian cancer patients. *Cancer*, **70**, 2851–2856.

ISHIWATA, I., ISHIWATA, C., SOMA, M., NOZAWA, S. & ISHIKAWA, H. (1987). Characterization of newly established human ovarian carcinoma cell line-special reference of the effects of cis-platinum on cellular proliferation and release of CA125. *Gynecol. Oncol.*, **26**, 340–354.

JOHNSON, V.G., SCHLOM, J., PATERSON, A.J., BENNETT, J., MAGNANI, J.L. & COLCHER, D. (1986). Analysis of a human tumor-associated glycoprotein (TAG-72) identified by monoclonal antibody B72.3. *Cancer Res.*, **46**, 850–857.

KANNAGI, R., FUKUSHI, Y., TACHIKAWA, T., NODA, A., SHIN, S., SHIGETA, K., HIRAIWA, N., FUKUDA, Y., INAMOTO, T., HAKOMORI, S. & IMURA, H. (1986). Quantitative and qualitative characterization of human cancer-associated serum glycoprotein antigens expressing fucosyl or sialyl-fucosyl type 2 chain polylactosamine. *Cancer Res.*, **46**, 2619–2626.

KIYOZUKA, Y. (1987). Establishment of human ovarian carcinoma cell line: characterization and tumor marker expression *in vitro*. *J. Nara Med.*, **38**, 459–479.

KJELDSEN, T., CLAUSEN, H., HIROHASHI, S., OGAWA, T., IIJIMA, H. & HAKOMORI, S. (1988). Preparation and characterization of monoclonal antibodies directed to the tumor-associated O-linked sialosyl 2-6 alpha-N-acetylgalactosaminyl (sialosyl-Tn) epitope. *Cancer Res.*, **48**, 2214–2220.

KLUG, T.L., BAST, R.C. Jr, NILOFF, J.M., KNAPP, R.C. & ZURAWSKI, V.R. Jr (1984). Monoclonal antibody immunoradiometric assay for an antigenic determinant (CA125) associated with human epithelial ovarian carcinomas. *Cancer Res.*, **44**, 1048–1053.

KUNIMATSU, M., ENDO, K., NAKASHIMA, T., AWAJI, T., SAGA, T., WATANABE, Y., KAWAMURA, Y., OHTA, H., KOIZUMI, M., SAKAHARA, H., KONISHI, J., FUJII, S., MORI, T., TORIZUKA, K., MATSUOKA, Y., NAKAGAMA, T. & YAMAGUCHI, N. (1988). Development of new immunoradiometric assay for CA125 antigen using two monoclonal antibodies produced by immunizing lung cancer cells. *Annals Nucl. Med.*, **2**, 73–79.

LAN, M.S., BAST, R.C. Jr, COLNAGHI, M.I., KNAPP, R.C., COLCHER, D., SCHLOM, J. & METZGAR, R.S. (1987). Co-expression of human cancer-associated epitopes on mucin molecules. *Int. J. Cancer*, **39**, 68–72.

MAGNANI, J.L., NILSSON, B., BROCKHAUS, M., ZOPF, D., STEPLEWSKI, Z., KOPROWSKI, H. & GINSBURG, V. (1982). A monoclonal antibody-defined antigen associated with gastrointestinal cancer is a ganglioside containing sialylated lacto-N-fucopentaose II. *J. Biol. Chem.*, **257**, 14365–14369.

MAGNANI, J.L., STEPLEWSKI, Z., KOPROWSKI, H. & GINSBERG, V. (1983). Identification of the gastrointestinal and pancreatic-cancer associated antigen detected by monoclonal antibody 19-9 in the sera of patients as a mucin. *Cancer Res.*, **43**, 5489–5492.

MASUHO, Y., ZALUTSKY, M., KNAPP, R.C. & BAST, R.C. Jr (1984). Interaction of monoclonal antibodies with cell surface antigens of human ovarian carcinomas. *Cancer Res.*, **44**, 2813–2819.

MATSUOKA, Y., NAKASHIMA, T., ENDO, K., YOSHIDA, T., KUNIMATSU, M., SAKAHARA, H., KOIZUMI, M., NAKAGAWA, T., YAMAGUCHI, N. & TORIZUKA, K. (1987). Recognition of ovarian cancer antigen CA125 by murine monoclonal antibody produced by immunization of lung cancer cells. *Cancer Res.*, **47**, 6335–6340.

MURARO, R., KUROKI, M., WUNDERLICH, D., POOLE, D.J., COLCHER, D., THOR, A., GREINER, J.W., SIMPSON, J.F., MOLINOLO, A., NOGUCHI, P. & SCHLOM, J. (1988). Generation and characterization of B72.3 second generation monoclonal antibodies reactive with tumor-associated glycoprotein 72 antigen. *Cancer Res.*, **48**, 4588–4596.

SAGA, T., ENDO, K., NAKASHIMA, T., AWAJI, T., KOIZUMI, M., KAWAMURA, Y., WATANABE, Y., KONISHI, J., NONOGAKI, H., NANBU, Y., FUJII, S. & MORI, T. (1990). Construction of an immunoradiometric assay for ovarian cancer associated antigen CA125 recognizing different antigenic determinant. *Acta Obstet. Gynecol. Scand.*, **69**, 175–181.

SAKAHARA, H., ENDO, K., NAKAJIMA, K., NAKASHIMA, T., KOIZUMI, M., OHTA, H., HIDAKA, A., KOHNO, S., NAKANO, Y., NAITO, A., SUZUKI, T. & TORIZUKA, K. (1986). Serum CA19-9 concentrations and computed tomography findings in patients with pancreatic carcinoma. *Cancer*, **57**, 1324–1326.

SAKAMOTO, M. (1988). Cyto-biological and immunological characterization of a newly established cell line (TMCC-1) derived from human uterine cervical adenocarcinoma. *J. Tokyo Med. Coll.*, **46**, 925–936.

SEARS, H.F., HERLYN, M., DEL VILLANO, B., STEPLEWSKI, Z. & KOPROWSKI, H. (1982). Monoclonal antibody detection of a circulating tumor-associated antigen. II. A longitudinal evaluation of patients with colorectal cancer. *J. Clin. Immunol.*, **2**, 141–149.

YU, Y.H., SCHLOSSMAN, D.M., HARRISON, C.L., RHINEHARDT-CLARK, A., SOPER, J.T., KLUG, T.L., ZURAWSKI, V.R. Jr & BAST, R.C. Jr (1991). Coexpression of different antigenic markers on moieties that bear CA125 determinants. *Cancer Res.*, **51**, 468–475.